# Factors Favoring Esophagectomy for Communicating Duplication Cysts

**DOI:** 10.1016/j.atssr.2025.05.002

**Published:** 2025-05-29

**Authors:** Kaylan N. Gee, Todd L. Demmy, Kenneth Patrick Seastedt

**Affiliations:** 1 Department of Surgery, University of Tennessee Graduate School of Medicine, Knoxville, Tennessee; 2 Department of Thoracic Surgery, Roswell Park Comprehensive Cancer Center, Buffalo, New York

## Abstract

A 54-year-old female patient presented with a symptomatic esophageal diverticulum. Endoscopy and biopsy confirmed a communicating esophageal duplication cyst with Barrett metaplasia and low-grade dysplasia. There was no evidence of fluorodeoxyglucose avidity on the preoperative positron emission tomography scan. Given recent trends towards esophageal preservation and absence of definitive cancer, cyst resection was planned. Final pathology revealed invasive adenocarcinoma; thus, esophagectomy was performed. We present a rare case of an esophageal duplication cyst progressing to invasive adenocarcinoma, focusing on the key surgical decision points in balancing oncologic control and esophageal preservation.

Esophageal duplication cysts are rare congenital malformations caused by foregut budding and vacuolization errors early in embryonic development, with a reported prevalence of 0.0122%.^1^ They are most often diagnosed in children, although occasionally discovered incidentally in adults (especially in the distal esophagus).^2^, ^3^, ^4^ More proximal cysts cause respiratory complaints; those in the lower esophagus incite dysphagia, emesis, and food impaction.^1^ Other intramural pathologies such as leiomyoma, lipoma, gastrointestinal stromal tumors, and esophageal malignancy serve as differential diagnoses.^3^ Historically, cyst resection was chosen to prevent complications like bleeding, infection, and perforation.^5^^,^^6^ A systematic review documented a shift from aggressive management by thoracotomy towards esophageal preservation with conservative management strategies like endoscopic intervention, minimally invasive resection, or expectant management.^4^ While malignant transformation of esophageal duplication cysts is rare, with only sporadic cases reported and the true incidence estimated to be exceptionally low,^2^^,^^4^^,^^7^ this risk must be assessed to inform treatment.

A 54-year-old female patient with a known 3 x 3 x 6 cm esophageal diverticulum (Figure 1) presented with symptoms of dysphagia, regurgitation, and aspiration. Upper endoscopy (Figure 2) confirmed a communicating esophageal duplication cyst with acid-induced Barrett metaplasia and low-grade dysplasia. There was no evidence of fluorodeoxyglucose avidity on a preoperative positron emission tomography/computed tomography (PET/CT) scan (Figure 3). Given the current trend favoring esophageal preservation in the absence of definitive cancer, cyst resection was planned rather than upfront esophagectomy.

**Figure 1:**
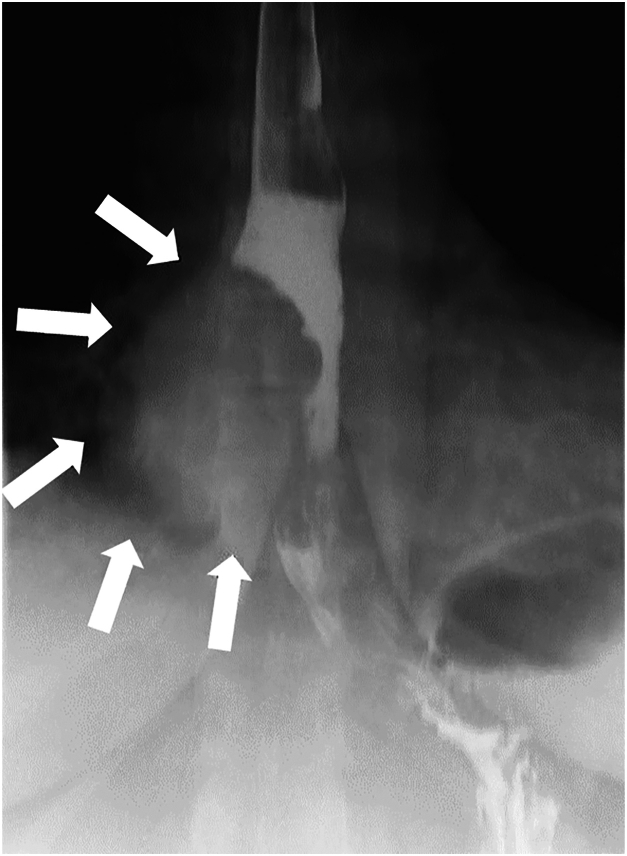
Barium esophagram demonstrating mucosal irregularity (arrows).

**Figure 2:**
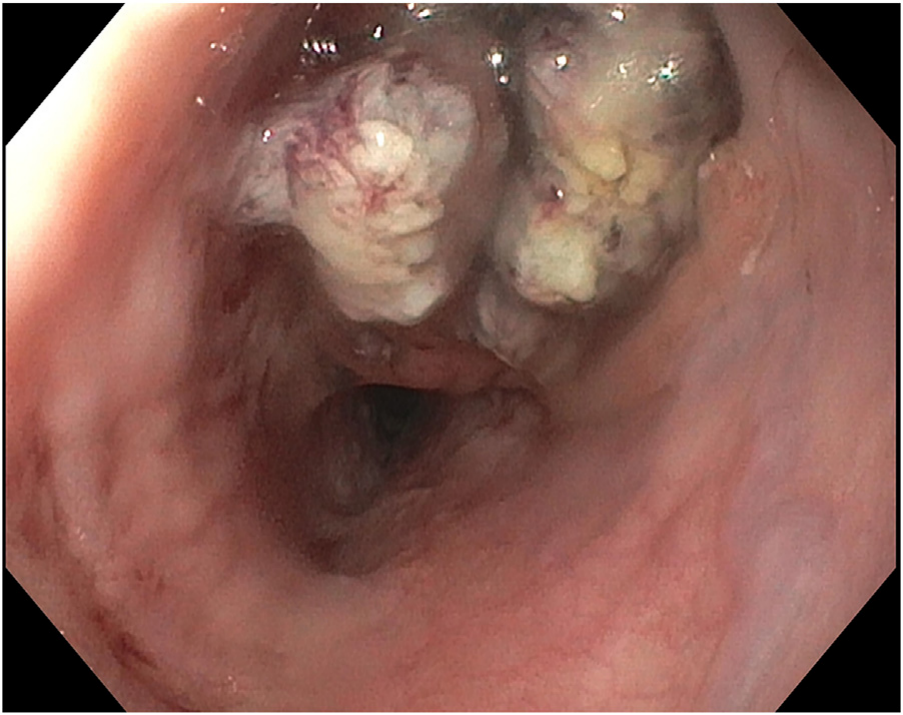
Upper endoscopy highlighting cyst communication with esophageal lumen.

**Figure 3:**
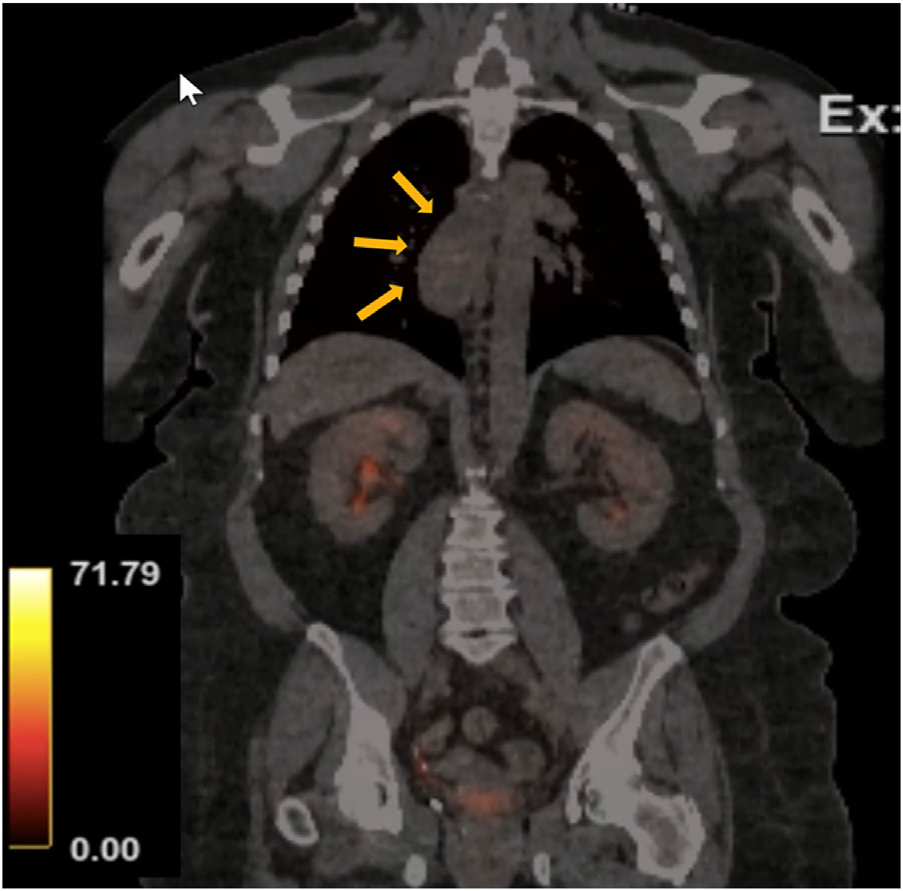
Preoperative positron emission tomography/computed tomography scan with no avidity (arrows highlighting irregularity).

The duplication cyst was exposed and opened robotically. Intraoperative biopsy revealed atypical cells attributed to the known dysplasia. The cyst communicated with approximately 30% of the true esophageal lumen circumference for 3-4 cm. The cyst wall was debrided, and an 8-cm mucosal defect was closed longitudinally with barbed suture and buttressed with residual esophageal muscularis propria to prevent free leakage. Final pathology revealed invasive adenocarcinoma. The mucosal repair dehisced asymptomatically on postoperative day 3 and luminal contents were contained by the muscular closure. On postoperative day 7, minimally invasive 3-field esophagectomy was performed for confirmed cancer. Esophagectomy pathology demonstrated a 0.5-cm pT2pN0 poorly differentiated adenocarcinoma with negative margins and 31 negative lymph nodes. The patient was discharged well on postoperative day 15 from the esophagectomy after conservative management of a subclinical cervical anastomotic leak. After multidisciplinary discussion and medical and radiation oncology consultations, surveillance was chosen for follow-up.

## Comment

This case demonstrates an opportunity for improved decision-making by offering immediate esophagectomy. The patient’s hospitalization was prolonged, in part due to the need for a repeat operation to address the previously undiagnosed malignancy.

Difficulty in widely sampling the cyst, a small tumor, and mucinous adenocarcinoma in a background of Barrett metaplasia contributed to the difficulty of identifying cancer preoperatively. Imaging used in the workup of these lesions typically includes barium esophagram, which can highlight mucosal irregularity and communication with the true esophageal lumen (80% do not connect).^3^ Endoscopic ultrasound further characterizes cyst contents, particularly distinguishing solid from cystic lesions, and defines tissue layers between cyst wall and surrounding structures.^1^^,^^6^ Cyst infection (up to 14% risk) results from endoscopic ultrasound with fine-needle aspiration (EUS-FNA).^3^^,^^6^ Accordingly, some authors avoid aspiration or advocate for the use of prophylactic antibiotics if EUS-FNA is used.^1^

EUS-FNA may be particularly useful in the workup of communicating cysts, which are more likely to demonstrate sequelae of acid exposure and may be less likely to develop infection due to their intrinsic ability to decompress into the esophagus. Depending upon cyst orientation, upper endoscopy and biopsy only under direct vision can be challenging. Thus, in the case of solid lesions or those with both solid and anechoic fluid components, EUS-FNA should be considered to augment diagnostic yield of tissue sampling, especially when lesions are concerning for malignancy.^1^^,^^3^

The utility of PET/CT in the preoperative workup could be argued. In this case, it was used to assess for unexpected malignancy when intervention was necessary due to increasing size and progressive dysphagia. Any evidence of organ invasion or distant metastasis on PET/CT would have shifted the treatment plan from surgery to nonoperative management. PET/CT is not typically recommended when evaluating early-stage esophageal cancer due to its limited utility in superficial tumors.^8^ In patients diagnosed with lesions confined to the submucosa using endoscopy and EUS, there is little correlation between fluorodeoxyglucose uptake and tumor depth. In a study of 79 patients with superficial tumors, there was 0% sensitivity for later demonstrated nodal disease.^8^ When PET/CT showed minimal fluorodeoxyglucose uptake, we engaged in shared decision-making with the patient and opted for cyst resection, knowing that esophagectomy may still be required.

Owing to the rarity of malignant transformation in esophageal duplication cysts, recommendations for esophagectomy must rely on clinician reasoning and an individualized risk assessment of patient factors. In our experience, large cyst to true lumen communication merits special consideration for planned esophagectomy. This particular feature may exacerbate esophageal symptoms from obstruction or substantial acid exposure. Florid Barrett metaplasia heightens the risk of progression to esophageal adenocarcinoma and its presence within the cyst may prompt upfront esophagectomy as surveillance becomes difficult and endoscopic intervention increasingly complex. While no precise threshold for troublesome cyst-true lumen communication exists, surgeon judgement—informed by the extent of epithelial injury—remains key in assessing cancer risk. Patients with notable symptoms, rapid cyst growth, and cysts that prevent safe reconstruction of the esophagus should also undergo planned esophagectomy. Liberal use of intraoperative biopsy can also decrease the need for repeat operations. Ultimately, complete resection is the only way to ensure the absence of malignancy.

This case highlights the nuanced decision-making involved in managing esophageal duplication cysts, particularly those with cyst-to-lumen communication and epithelial transformation. While malignant transformation within these rare cysts is well documented, this case illustrates how anatomic communication, acid-induced epithelial injury, and diagnostic limitations can mask early invasive adenocarcinoma. Our experience suggests that cysts with large communications to the true esophageal lumen should heighten suspicion for malignancy and may justify planned esophagectomy over cyst resection, even without definitive preoperative evidence of cancer. Beyond adding to the limited reports documenting malignant transformation, this case underscores the limits of imaging, endoscopy, and intraoperative biopsy in excluding cancer. It also reinforces the importance of shared decision-making, including explicit counseling that reoperation may be necessary if final pathology reveals malignancy. Together, these insights support a more aggressive surgical strategy, informed by case-specific anatomic considerations and patient preferences, when managing rare esophageal lesions with uncertain malignant potential.
